# Assessment of Village‐Based Chicken Production System in East Gojam Zone, Ethiopia

**DOI:** 10.1155/vmi/1557614

**Published:** 2026-05-29

**Authors:** Dessie Abera Wondie, Mezgebu Getnet

**Affiliations:** ^1^ Animal Health Research Program, Ethiopian Institute of Agricultural Research, Debre Markos Research Center, Debre Markos, Ethiopia, eiar.gov; ^2^ Animal Feed and Nutrition Research Program, Ethiopian Institute of Agricultural Research, Debre Markos Research Center, Debre Markos, Ethiopia, eiar.gov

**Keywords:** assessment, East Gojam, production, village chicken

## Abstract

The study was conducted to assess chicken handling practices and identify the major challenges affecting village‐based chicken production in the East Gojam zone, Ethiopia. The study sites were selected randomly, while households were purposively selected based on their experience in chicken production and residence in rural areas. A cross‐sectional study design was employed, and a standardized questionnaire was administered to 86 selected households. Data were analyzed using SPSS Version 20 software. The average flock size per household was 12.06 chickens, with 71.4% of them being indigenous breeds, and all were free scavengers. Almost all respondents supplemented their chickens with homemade grains and leftovers, although this was not adequate. Hens dominated the flock, and the cock‐to‐hen ratio was 1:3.97. Most respondents (52.3%) restocked their chickens by hatching at home. The major constraints of chicken production identified were diseases, predator attacks, poor hatchability, and a shortage of feed resources. Newcastle disease (ND) occurred in 54.1% of the households, and its occurrence was statistically associated with study woredas (*χ* ^2^ = 13.406, *p* value = 0.009). It caused high chicken mortality in the area. However, most of the respondents did not practice disease control and prevention biosecurity measures. Approximately 68.6% of households did not use the ND vaccine, 44.2% mixed newly purchased chickens with their own without quarantine, 71.4% allowed their chickens to have contact with neighboring flocks, and most households disposed of dead chickens outside their compounds, which may promote disease spread. Predators affected 78.6% of the household chickens. Therefore, it is essential to create awareness about proper chicken handling, vaccination, and implementing biosecurity measures to enhance the productivity and sustainability of village chicken production.

## 1. Introduction

Poultry is the largest group of livestock species worldwide [1] and contributes about 30% of all animal protein consumed globally; however, a shortage of protein availability is a well‐known problem in Africa [2]. The total poultry population in Ethiopia is estimated to be about 41.38 million, and 96% of them are raised under the traditional backyard management system, while the remaining 4% are within the intensive management system [2, 3]. Various advantages make poultry production attractive in poverty alleviation and quality protein supply in Sub‐Saharan Africa [3] because of short generation intervals, a high rate of productivity, the ease with which products can be supplied to different areas, and the ease with which products can be sold because of their relatively low economic value [1]. Backyard chicken production is a sustainable livelihood option for millions in rural communities. They have been important for over eight thousand years since their domestication. Rearing backyard chickens requires minimal inputs and is affordable for resource‐limited households. They contribute significantly to poverty alleviation and household food security, ensure environmental sustainability (pest control and manure provision), and can support HIV/AIDS mitigation and wildlife conservation initiatives [4–6]. Furthermore, backyard chicken production is very important for women [7]. It provides women with an immediate income to meet household expenses and sources of food [8]. Therefore, they have the potential to help achieve the development goals of a country [4].

However, the major challenges of backyard chicken farming are limited access to improved agricultural technologies, poor access to veterinary services, predation, the occurrence of infectious diseases, poor biosecurity practices, poor housing and feeding, inadequate training on scientific management practices, marketing problems, and inadequate agricultural credit [9–11]. The main problems of indigenous chicken production in the tropics are low hatchability (at about 70%), high chick mortality, and longer reproductive cycles. During the first eight weeks of life, 40%–60% of chicks die, mostly from disease and predator attacks. The traditional poultry production system is characterized by the periodic devastation of the flock by disease [8, 12]. However, disease prevention interventions involving vaccination of backyard chickens in India against infectious diseases have been demonstrated to have a dramatic impact on the survival of the chickens and on household food security and profitability [13]. In Ethiopia, the village‐based chicken production is characterized by few inputs for housing, feeding, and healthcare, along with a high level of mortality. Scavenging is the only source of diet [11]. The demand for the nation’s food supply increases with population growth. As a result, there will be a significant increase in demand for animal products [2]; under this condition, the demand for eggs and chicken meat for the Ethiopian population cannot be satisfied [14].

The most important inputs to satisfy the demand for poultry products are introducing improved breeds, providing quality feeds, vaccines, and medicaments [15]. Different exotic poultry breeds were introduced to smallholder farming systems in Ethiopia to improve poultry productivity. However, their contribution to the Ethiopian economy is significantly lower than that of other African countries [2]. This is because of poor management practices and different poultry production constraints, such as diseases, feed shortages, predators, and lack of veterinary services. Poultry diseases are the primary challenge for Ethiopian poultry production [16]. The majority of Ethiopia’s poultry population is raised under the traditional backyard management system. They are ideal for rural development efforts due to their adaptability to local resources and minimal reliance on external inputs. Thus, identifying the constraints present in the village‐based production system is essential in improving chicken production to enhance smallholder farmers’ livelihoods and the sustainable development of the country. Although the village chicken production system has been widely described by many scholars in Ethiopia, information on the backyard chicken management and biosecurity practices at the household level in the East Gojam zone is limited. Therefore, the objectives of this study were to assess chicken handling practices and biosecurity measures practiced and to identify the major challenges affecting village chicken production in the study area.

## 2. Materials and Methods

### 2.1. Study Area Description

**FIGURE 1:**
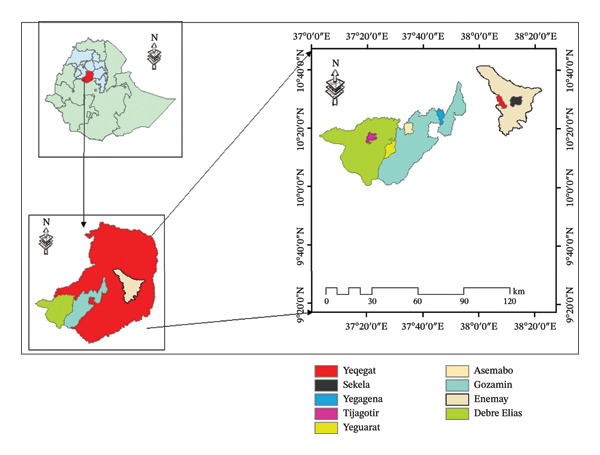
Map of the study area.

The study was conducted in the Enemay, Gozamin, and Debre Elias woredas of the East Gojam zone (Figure 1). East Gojam is one of the administrative zones of the Amhara National Regional State (ANRS) located in the Blue Nile Basin of Ethiopia. It is structured with 16 woredas and four urban administrations. The majority of the population (86.5%) in the zone lives in rural areas where agriculture is the main source of livelihood [17]. The East Gojam zone covers different topographic features with an elevation ranging from 800 to 4200 m.a.s.l. The rainfall pattern is mainly of the unimodal type, and the average annual rainfall varies from 900 to 1800 mm. The average temperature ranges from a minimum of 7.5°C to a high of 27°C. It is one of the major potential areas for livestock production, and it has a large number of different livestock species that account for a significant proportion of the ANRS livestock resource [18]. The total population of poultry at the country level is estimated to be about 41.35 million. Regarding breed, 78.04%, 17.58%, and 4.34% of the total poultry in the country; 83.2%, 10.8%, and 6.0% of the poultry in the Amhara region; and 80.0%, 17.7%, and 2.3% of the poultry in the East Gojam zone were indigenous, hybrid, and exotic, respectively [19]. The selected woredas for the study in the East Gojam administrative zone have the potential for poultry production and have a relatively high poultry population. Indigenous chickens are essential for the livelihood of smallholder farmers, and they make up the largest percentage [20].

### 2.2. Study Design and Sampling Methods

A cross‐sectional study was conducted from October 2024 to June 2025, and a standardized questionnaire was prepared and administered to 86 chicken producers to assess their experience in chicken handling, biosecurity practices, and disposal of manure and dead chicken. The three woredas and two kebeles from each woreda were selected using a simple random sampling technique. However, 15 poultry keepers from each kebele were selected purposively in consultation with kebele animal health technicians based on their experience in chicken production, residence in rural areas, accessibility, and willingness to participate. These eligibility criteria were determined to ensure the inclusion of active poultry‐keeping households in the respective study kebeles to meet the objectives of the study. The chicken production experience of the household for inclusion in this study was determined based on ownership of village chickens or the availability of chickens in their household. Regarding sample size, two kebeles from every three woredas and 15 households from every kebele would be a total of 90 households. However, the interview results of four respondents were incomplete, and their data were excluded from the analysis.

### 2.3. Ethical Statement

The study involved a noninvasive data collection method, which consisted of questionnaire‐based interviews with the selected volunteer householders. The study was carried out according to internationally accepted ethical guidelines for research involving human participants, as outlined by the World Health Organization and the Council for International Organizations of Medical Sciences. Before the questionnaire survey was conducted, all participants were given an in‐depth description of the purpose of the study, and verbal informal consent was obtained. Participation of the householders in this study was totally voluntary, and confidentiality of the information was strictly maintained. Moreover, no personal identifiers were recorded.

### 2.4. Data Analysis

The collected data were recorded properly, entered into a Microsoft Excel spreadsheet, and coded. Then, descriptive statistics, one‐way ANOVA, and chi‐square tests were used to summarize the data by the SPSS software package to calculate frequencies and percentages and to analyze the association between different parameters. Confidence level is at 95%, and a value of *p* < 0.05 was considered statistically significant.

## 3. Results

### 3.1. Respondents’ Profile

Eighty‐six household respondents participated in the study, and about 53 (61.6%) were male, and the rest were female. The educational status and sex of participants are summarized in Table 1.

**TABLE 1:** Socioeconomic characteristics of respondents.

Variables		Frequency	Percent
Sex	Male	53	61.6
	Female	33	38.4

Educational level	No formal education	30	35.7
	Primary school	36	42.9
	Secondary school	17	20.2
	College diploma and above	1	1.2

### 3.2. Chicken Flock Size

The total flock size was 1,037, and on average, each respondent had 12.06 chickens, with a range of 3–42 chickens. The flock size was relatively highest in Debre Elias, but the highest percentage of local chicken was from Enemay woreda. The flock size in each woreda from the selected respondents is described in Table 2. In total, the cock‐to‐hen ratio was 1:3.97, and the flock was dominated by hens, which were followed by chicks. However, it was higher in Debre Elias (1:6.5) compared to other study woredas. The chickens available in the selected households were categorized as indigenous (local chickens), exotic (Sasso and Koekoek breed chickens), and crossbred chickens. Overall, 71.4% of the chickens in the area were indigenous chickens and the highest (90%) was from Enemay woreda compared with others.

**TABLE 2:** Respondents’ flock size.

Woredas	Chicken categories	Flock size < 2 months	Flock size 2–6 months	Cocks	Hens	Total	Indigenous (%)	Cock:hen ratios
Gozamin	Indigenous	40	103	39	89	271	77	1:2.28
	Exotic	8	28	12	21	69		1:1.75
	Crossbred	0	6	2	4	12		1:2
	Total	48	137	53	114	352		1:2.15

Enemay	Indigenous	7	76	24	67	174	90.2	1:2.79
	Exotic	0	5	3	11	19		1:3.66
	Crossbred	0	0	0	0	0		0
	Total	7	81	27	78	193		1:2.88

Debre Elias	Indigenous	46	50	38	161	295	60	1:4.23
	Exotic	5	16	12	164	197		1:13.66
	Crossbred	0	0	0	0	0		0
	Total	51	66	50	325	492		1:6.5

Total		106	284	130	517	1037		1:3.97

### 3.3. Chicken Handling Practices

The chicken‐rearing system practiced in the area was based on a traditional production system. About 36.5%, 32.9%, and 25.9% of the households considered all family members, children, and women for the chicken‐handling responsibilities, respectively. Approximately 52.3% of the respondents restock their chickens by hatching at home. Chickens were confined at night only, and 25.6% of the respondents’ chickens shared the main house (Table 3). About 93% of the respondents clean the chickens’ house every day. The reason for the remaining respondents who did not clean the chicken house every day was due to workload and being busy (four out of six respondents), and the remaining were due to lack of awareness, and the house was wide enough and had no fecal accumulation. Regarding manure disposal, most of the respondents used it as a fertilizer (67.4%), threw it out of a compound (13.95%), and buried it (18.6%).

**TABLE 3:** Chicken management and restocking practices.

Variables		Gozamin	Enemay	Debre Elias	Total (%)	*χ* ^2^ (*p* value)
House cleaning practices	Every day	30 (100.0)	24 (92.3)	26 (86.7)	80 (93.0)	4.138 (0.126)
	Every 3 days	0 (0.0)	1 (3.8)	4 (13.3)	5 (5.8)	
	Weekly	0 (0.0)	1 (3.8)	0 (0.0)	1 (1.2)	

Do you mix purchased with your own chicken?	Yes	19 (63.3)	14 (53.8)	5 (16.7)	38 (44.2)	27.783 (0.000)
	No	10 (33.3)	7 (26.9)	25 (83.3)	42 (48.8)	
	NA	1 (3.3)	5 (19.2)	0 (0.0)	6 (7.0)	

Purchased chicken handling	Separate for one day	0 (0.0)	1 (14.3)	2 (13.3)	3 (11.1)	13.202 (0.213)
	Separate for 2–3 days	3 (60.0)	4 (57.1)	10 (66.7)	17 (63.0)	
	Separate for 4–5 days	1 (20.0)	2 (28.6)	2 (13.3)	5 (18.5)	
	Separate for a week	1 (20.0)	0 (0.0)	0 (0.0)	1 (3.7)	
	Separate for 2 weeks	0 (0.0)	0 (0.0)	1 (6.7)	1 (3.7)	

Contact with neighboring flocks	Yes	22 (78.6)	16 (61.5)	22 (73.3)	60 (71.4)	5.822 (0.213)
	No	6 (21.4)	10 (38.5)	8 (26.7)	24 (28.6)	

Where do you get chicken for restocking?	Local market	6 (20.0)	6 (23.1)	3 (10.0)	15 (17.4)	18.454 (0.048)
	Commercial farms	0 (0.0)	0 (0.0)	2 (6.7)	2 (2.3)	
	Hatching at home	12 (40.0)	11 (42.3)	22 (73.3)	45 (52.3)	
	Buy fertile eggs and hatching at home	2 (6.7)	1 (3.8)	0 (0.0)	3 (3.5)	
	Local market and hatching at home	10 (33.3)	8 (30.8)	2 (6.7)	20 (23.3)	
	Purchasing from known neighbors	0 (0.0)	0 (0.0)	1 (3.3)	1 (1.2)	

Chicken housing at night?	Kitchen and/or store	0 (0.0)	2 (7.7)	0 (0.0)	2 (2.3)	13.863 (0.085)
	Separate chicken house	19 (63.3)	17 (65.4)	22 (73.3)	58 (67.4)	
	In the main house	9 (30.0)	5 (19.2)	8 (26.7)	22 (25.6)	
	Perch on trees	2 (6.7)	0 (0.0)	0 (0.0)	2 (2.3)	
	Perch in the cattle barn	0 (0.0)	2 (7.7)	0 (0.0)	2 (2.3)	

In this study, approximately 44.2% of households mix purchased chickens with their own chickens in the first days without quarantine. There were statistically significant associations between study woredas (*χ* ^2^ = 27.783, *p* value = 0.000), and those who separate purchased chickens, almost all of them quarantine the chickens for less than a week. All the chickens available in the study area are free scavengers, and about 71.4% of the household chickens have contact with other flocks.

### 3.4. Respondents’ Awareness About Newcastle Disease (ND)

Most of the respondents (90.7%) have heard of ND, and it has occurred in 54.1% of the respondents’ chicken farms. The occurrence of ND was statistically associated with study woredas (*χ* ^2^ = 13.406, *p* value = 0.009). All age groups (53.3%) were affected. Contact with neighboring household chickens (42.9%) and incoming chickens from markets (unsold or purchased) (20.4%) were considered as the main suspected source of infection (Table 4). About 90.6% of the respondents considered the ND as a concerning disease in chicken production. However, 68.6% of the households did not use the ND vaccine, and 18.8% of the respondents did not know that the spread of the disease between chickens can be prevented. The main reason for not vaccinating their chicken against ND was a lack of awareness (65.5%). From the respondents who got chicken infection in that year, 52.3% of the households responded that ND was the disease that occurred at their farm, whereas 47.7% of them did not know the disease encountered. The disease occurrence was statistically significant (*χ* ^2^ = 33.46, *p* value = 0.000) in Gozamin compared to other study woredas. The average number of chickens that died in the last year per household ranges from 2 to 36. Complete mortality of affected chickens occurred in about 40% of the households. Dead birds were thrown out of a compound (45.2%), buried (41.7%), thrown into a river (3.6%), eaten by dogs and cats (6%), or thrown into garbage or a hole (3.6%).

**TABLE 4:** Respondents’ Newcastle disease awareness.

Variables		Woreda			Total	*χ* ^2^ (*p* value)
		Gozamin	Enemay	Debre Elias		
Have you ever heard of Newcastle disease?	Yes	30 (100.0)	25 (96.2)	23 (76.7)	78 (90.7)	10.995 (0.004)
	No	0 (0.0)	1 (3.8)	7 (23.3)	8 (9.3)	

Have you experienced ND in your flock?	Yes	21 (72.4)	16 (61.5)	9 (30.0)	46 (54.1)	13.406 (0.009)
	No	8 (27.6)	10 (38.5)	21 (70.0)	39 (45.9)	

Age groups affected	< 6 months of age	5 (33.3)	3 (25.0)	0 (0.0)	8 (26.7)	13.701 (0.033)
	All age groups	7 (46.7)	7 (58.3)	2 (66.7)	16 (53.3)	
	All age groups (severe in layers and growers)	3 (20.0)	2 (16.7)	1 (33.3)	6 (20.0)	

The proportion of sick chickens that died	All	5 (23.8)	3 (100.0)	4 (66.7)	12 (40.0)	34.017 (0.000)
	Two‐third	6 (28.6)	0 (0.0)	1 (16.7)	7 (23.3)	
	Half	4 (19.0)	0 (0.0)	0 (0.0)	4 (13.3)	
	One‐third	2 (9.5)	0 (0.0)	1 (16.7)	3 (10.0)	
	Quarter	4 (19.0)	0 (0.0)	0 (0.0)	4 (13.3)	
	Total	21 (100.0)	3 (100.0)	6 (100.0)	30 (100.0)	

Suspected source of infection	Incoming chicken from the market/unsold or purchased	5 (22.7)	4 (25.0)	1 (9.1)	10 (20.4)	24.763 (0.037)
	Contact with the neighboring household’s chicken	11 (50.0)	5 (31.3)	5 (45.5)	21 (42.9)	
	Chicken trader visit	1 (4.5)	0 (0.0)	0 (0.0)	1 (2.0)	
	Exchange cock	1 (4.5)	0 (0.0)	0 (0.0)	1 (2.0)	
	Contamination by scavengers	1 (4.5)	0 (0.0)	3 (27.3)	4 (8.2)	
	I do not know	3 (13.6)	7 (43.7)	2 (18.2)	12 (24.5)	

Do you agree that ND is a concerning disease?	Yes	29 (96.7)	25 (100.0)	23 (76.7)	77 (90.6)	13.077 (0.011)
	No	1 (3.3)	0 (0.0)	7 (23.3)	8 (9.4)	

Can the spread of the disease among chickens be prevented?	Yes	23 (79.3)	22 (84.6)	23 (76.7)	68 (80.0)	4.444 (0.617)
	No	5 (17.2)	4 (15.4)	7 (23.3)	16 (18.8)	
	No idea	1 (3.5)	0 (0.0)	0 (0.0)	1 (1.2)	

Have you ever used the ND vaccine?	Yes	12 (40.0)	6 (23.1)	9 (30.0)	27 (31.4)	1.894 (0.388)
	No	18 (60.0)	20 (76.9)	21 (70.0)	59 (68.6)	

Why did not you vaccinate your chicken against ND?	Lack of awareness	13 (72.2)	13 (68.4)	12 (57.1)	38 (65.5)	16.307 (0.038)
	Chickens do not need a vaccine	3 (16.7)	0 (0.0)	0 (0.0)	3 (5.2)	
	Poor access to vaccination points	2 (11.1)	4 (21.1)	2 (9.5)	8 (13.8)	
	No ND outbreak	0 (0.0)	2 (10.5)	7 (33.3)	9 (15.5)	

Do you feel that vaccination can prevent the disease?	Yes	23 (76.7)	20 (76.9)	22 (75.9)	65 (76.5)	6.965 (0.324)
	No	2 (6.7)	5 (19.2)	2 (6.9)	9 (10.6)	
	No idea	5 (16.7)	1 (3.8)	5 (17.2)	11 (12.9)	

### 3.5. Chicken Infection and the Measures Taken

The majority of households (93%) access veterinary services. However, only 44.8% of the respondents isolate their sick chickens and look for veterinary services. The measures taken following a chicken disease outbreak in their village were confining at home (39.5%), trying preventive treatment (37.2%), and selling (18.6%) (Table 5). About 68% of the respondents treated all their chickens, while the remaining respondents treated only sick chickens. Fourteen percent of the respondents treated their chickens by themselves. About 26.7% of the households use medical drugs for their chicken treatment. The medical drugs used were ampicillin (60.9%) and tetracycline (30.4%), while 8.7% of the respondents used both drugs. The route of administration was through feed (39.1%) and drinking water (47.8%). The major reasons for using medical treatments for chickens were that they could cure the disease (60%), treat them at home until they could be taken to a veterinary clinic (15%), and serve as an alternative when veterinary clinics were closed on weekends (10%). The highest disease occurrence was observed during the summer (42.7%) compared to other seasons. About 41.7% of the respondents bury dead chickens as a biosecurity measure.

**TABLE 5:** Chicken disease and the measures taken.

Variables		Gozamin	Enemay	Debre Elias	Total
Access to veterinary service	Yes	27 (90.0)	25 (96.2)	28 (93.3)	80 (93.0)
	No	3 (10.0)	1 (3.8)	2 (6.7)	6 (7.0)

What do you do when you hear chicken disease outbreak in your village?	Sell	9 (30.0)	4 (15.4)	3 (10.0)	16 (18.6)
	Try preventive treatment	14 (46.7)	11 (42.3)	7 (23.3)	32 (37.2)
	Confine at home	6 (20.0)	9 (34.6)	19 (63.3)	34 (39.5)
	Do nothing	1 (3.3)	2 (7.7)	1 (3.3)	4 (4.7)

What do you do when chickens get sick?	Isolate from healthy chicken	5 (17.2)	4 (15.4)	4 (13.3)	13 (15.3)
	Look for veterinary services	12 (41.4)	4 (15.4)	3 (10.0)	19 (22.4)
	Treat with traditional medicine	5 (17.2)	1 (3.8)	3 (10.0)	9 (10.6)
	Sell	2 (6.9)	0 (0.0)	0 (0.0)	2 (2.4)
	Isolate and look for vet services	2 (6.9)	10 (38.4)	7 (23.3)	19 (22.4)
	Isolate and use traditional medicines	2 (6.9)	5 (19.2)	11 (36.7)	18 (21.2)
	Do nothing	1 (3.4)	2 (7.7)	2 (6.7)	5 (5.9)

Which one do you treat?	All your chicken	21 (80.8)	5 (41.7)	8 (66.7)	34 (68.0)
	Only sick chicken	5 (19.2)	7 (58.3)	4 (33.3)	16 (32.0)

Who administers the treatment?	Yourself	7 (28.0)	0 (0.0)	0 (0.0)	7 (14.0)
	Animal health personnel	18 (72.0)	13 (100.0)	12 (100.0)	43 (86.0)

Dead chicken disposal	Buried	9 (30.0)	10 (40.0)	16 (55.2)	35 (41.7)
	Thrown into the garbage or whole	1 (3.3)	2 (8.0)	0 (0.0)	3 (3.6)
	Eaten by dogs and cats	2 (6.7)	2 (8.0)	1 (3.4)	5 (6.0)
	Thrown out of a compound	15 (50.0)	11 (44.0)	12 (41.4)	38 (45.2)
	Thrown into a river	3 (10.0)	0 (0.0)	0 (0.0)	3 (3.6)

Which season do you observe high disease occurrence?	Summer	18 (64.3)	7 (30.4)	7 (29.2)	32 (42.7)
	Winter	2 (7.1)	5 (21.7)	4 (16.7)	11 (14.7)
	Spring	5 (17.9)	9 (39.1)	9 (37.5)	23 (30.7)
	Summer and spring	2 (7.1)	1 (4.3)	4 (16.7)	7 (9.3)
	Winter and spring	1 (3.6)	1 (4.3)	0 (0.0)	2 (2.7)

ND has occurred in chicken farms of approximately 54.1% of the respondents (Table 4). The chicken management practices that might be associated with the occurrence of the disease in each household are described in Table 6. The occurrence of ND was statistically associated with the disposal practices of dead chickens, contact with neighboring household flocks, and mixing of purchased with their own chickens (*p* value < 0.05). This indicates that poor biosecurity practices may be associated to the transmission of ND virus in the area.

**TABLE 6:** The chicken management practices and the occurrence of ND in the selected households.

The management practices and study sites		The occurrence of the ND in households’ flock		*χ* ^2^ (*p* value)
		Yes	No	
House cleaning every day	Yes	44 (55.7)	35 (44.3)	1.211 (0.546)
	No	2 (33.3)	4 (66.7)	

Dead chicken disposal	Buried	11 (31.4)	24 (68.6)	44.862 (0.000)
	Thrown into the garbage or whole	2 (66.7)	1 (33.3)	
	Eaten by dogs and cats	4 (80.0)	1 (20.0)	
	Thrown out of a compound	27 (71.1)	11 (28.9)	
	Thrown into a river	2 (100.0)	0 (0.0)	

Contact with neighboring flocks	Yes	40 (67.8)	19 (32.2)	20.294 (0.000)
	No	4 (16.7)	20 (83.3)	

Mixing of purchased with their own chickens	Yes	28 (73.7)	10 (26.3)	12.042 (0.017)
	No	15 (36.6)	26 (63.4)	
	No idea	3 (50.0)	3 (50.0)	

Study woredas	Gozamin	21 (72.4)	8 (27.6)	13.406 (0.09)
	Enemay	16 (61.5)	10 (38.5)	
	Debre Elias	9 (30.0)	21 (70.0)	

Chicken restocking practices	Local market	12 (80.0)	3 (20.0)	12.266 (0.268)
	Commercial farms	2 (100.0)	0 (0.0)	
	Hatching at home	17 (38.6)	27 (61.4)	
	Buy fertile eggs and hatching at home	2 (66.7)	1 (33.3)	
	Local market and hatching at home	12 (60.0)	8 (40.0)	
	Purchasing from known neighbors	1 (100.0)	0 (0.0)	

### 3.6. Chicken Feed and Feed Scarcity in the Area

Village chickens in the study area were primarily maintained under a scavenging feeding system. However, respondents offer supplementary feeds in addition to scavenging. Almost all of the respondents supplement their chicken with homemade grains and leftovers, even though this is not adequate. Feed scarcity was aggravated in the wet season (90.5%) compared to the dry season (Table 7). The major coping mechanisms practiced for feed scarcity were giving feed from human consumption (50.6%), selling to decrease their number (18.1%), and providing feed for the remaining chickens (26.5%).

**TABLE 7:** Season of chicken feed scarcity and coping mechanisms.

Variables	Gozamin	Enemay	Debre Elias	Total
*The season of feed scarcity aggravated*				
Wet	27 (90.0)	20 (83.3)	29 (96.7)	76 (90.5)
Dry	3 (10.0)	3 (12.5)	0 (0.0)	6 (7.1)
Both	0 (0.0)	1 (4.2)	1 (3.3)	2 (2.4)

*Coping mechanism for feed scarcity*				
Selling to decrease their number	12 (41.4)	1 (4.2)	2 (6.7)	15 (18.1)
Giving feed from human consumption	2 (6.9)	19 (79.2)	21 (70.0)	42 (50.6)
Selling them and giving feed	13 (44.8)	3 (12.5)	6 (20.0)	22 (26.5)
Making insects and termites available	2 (6.9)	0 (0.0)	0 (0.0)	2 (2.4)
No feed problem	0 (0.0)	1 (4.2)	1 (3.3)	2 (2.4)

The major grains supplemented to the chicken were wheat (96.5%) and maize (82.6%) (Table 8). Even though feed scarcity was aggravated in the wet season, 48.2% of the respondents supplemented their chickens in all seasons of the year. About 86.9% of the respondents supplement their chicken in the morning and late afternoon. All respondents from Enemay and Debre Elias evaluated their chicken after supplementation. The reason for supplementation was to improve egg production (65.7%) and growth and egg production (27.1%). The highest percentage (51.8%) of households use water wells as a source of water for their chickens. About 84.9% of the households were interested in expanding their poultry farms and that was mainly to get better income (73.5%). However, the remaining 15.1% of the respondents are not interested in expanding their poultry farms. This was due to a lack of awareness (36.4%), labor shortages (27.3%), feed problems (27.3%), and damage to horticultural crops by chickens (9.1%).

**TABLE 8:** Common chicken feed supplements practiced in the study area.

Variables	Gozamin	Enemay	Debre Elias	Total
*Grains used (%)*				
Maize	60.0	96.2	93.3	82.6
Wheat	100.0	88.5	100.0	96.5
Sorghum	46.7	3.8	0.0	17.4
Barely	20.0	7.7	0.0	9.3
Oats	6.7	0.0	0.0	2.3

*Supplementation season (%)*				
All seasons	53.3	40.0	50.0	48.2
Wet season	33.3	44.0	50.0	42.4
Dry season	13.3	16.0	0.0	9.4

*Supplementation time (%)*				
Morning	10.3	0.0	20.0	10.7
Late afternoon	3.4	0.0	3.3	2.4
Morning and late afternoon	86.2	100.0	76.6	86.9

*Those who evaluate their chicken after supplementation? (%)*				
	46.7	100.0	100.0	81.4

*The reason for supplementation (%)*				
For continuous laying	92.9	46.2	70	65.7
For fast growth and continuous laying	7.1	38.5	26.7	27.1
For fast growth and early laying	0.0	15.4	0.0	5.7
To strengthen egg shell	0.0	0.0	3.3	1.4

*Water source (%)*				
Tap water	17.2	26.9	0.0	14.1
Water wells	58.6	46.2	50.0	51.8
River	17.2	11.5	6.7	11.8
Pond	6.9	0.0	26.7	11.8
Hand pump	0.0	15.4	16.7	10.6

*Those who are interested for expanding their farm (%)*				
	83.3	84.6	86.7	84.9

*Why they are interested for expanding? (%)*				
To get a better income	80.0	68.2	76.9	75.3
For cash income and home consumption	12.0	13.6	7.7	11.0
Eggs and chicken are becoming expensive	0.0	13.6	11.5	8.2
It is not laborious	8.0	4.5	3.8	5.5

### 3.7. Common Predators in the Area

About 78.6% of household chickens were affected by predators. Wildcats and eagles were the major predators in the area (Table 9).

**TABLE 9:** Common chicken predators in the area.

Variables		Gozamin	Enemay	Debre Elias	Total
Is there predator loss in your chicken?	Yes	23 (79.3)	22 (84.6)	21 (72.4)	66 (78.6)
	No	6 (20.7)	4 (15.4)	8 (27.6)	18 (21.4)

Common predators in your area	Wildcat	8 (32.0)	16 (72.7)	8 (44.4)	32 (49.2)
	Eagle	0 (0.0)	3 (13.6)	2 (11.1)	5 (7.7)
	Wildcat and eagle	15 (60.0)	3 (13.6)	4 (22.2)	22 (33.9)
	Domestic cat	2 (8.0)	0 (0.0)	2 (11.1)	4 (6.2)
	Foxes	0 (0.0)	0 (0.0)	1 (5.6)	1 (1.5)
	Human	0 (0.0)	0 (0.0)	1 (5.6)	1 (1.5)

### 3.8. Constraints of Chicken Production

In the current study, disease, predator attack, poor hatchability, and shortage of feed resources were ranked first (Table 10). However, from these constraints, poultry disease and predator attacks were the major constraints identified in the village chicken production of the study area.

**TABLE 10:** Constraints of chicken production in the household.

Variables	Frequency (%) (*N* = 86)					
	1^st^ rank	2^nd^ rank	3^rd^ rank	4^th^ rank	5^th^ rank	6^th^ rank
Shortage of feed resources	6 (7.0)	6 (7.0)	8 (9.3)	6 (7.0)	2 (2.3)	—
Disease occurrence	33 (38.4)	11 (12.8)	5 (5.8)	6 (7.0)	—	—
Predator attack	14 (16.3)	28 (32.6)	2 (2.3)	1 (1.2)	2 (2.3)	—
Poor hatchability	9 (10.5)	5 (5.8)	15 (17.4)	5 (5.8)	1 (1.2)	1 (1.2)
Poor access to the market	1 (1.2)	3 (3.5)	4 (4.7)	2 (2.3)	1 (1.2)	2 (2.3)
Space of rearing/production	4 (4.7)	—	1 (1.2)	—	—	—
Spoilage of eggs	—	6 (7.0)	9 (10.5)	6 (7.0)	4 (4.7)	3 (3.5)

## 4. Discussion

In this study, the proportion of female respondents (38.4%) who participated in the interview was relatively higher compared with other reports from different parts of Ethiopia; for instance, 74.6% and 25.4% and 73.6% and 26.4% of the interviewed smallholder farmers were male and female, respectively, in Northwest and Western Ethiopia [16, 21]. Regarding the educational status of respondents, about 35.7% of them had no formal education. This finding is comparable with the report of Jimma et al., where about 37% of the respondents in Southern Ethiopia were illiterate [22]. Each respondent owned an average of 12.06 chickens, with flock sizes ranging from 3 to 42 chickens. This was comparable with the report of Chaka et al. from East Shewa, where the flock size ranged from 1 to 36 [23]. The average flock size of this study (12.06) per household agreed with the report of Moges et al., where the average flock size per household was 13 [21]. In other reports, the average flock size was 10 and 7.76 per household from the Benishangul‐Gumuz region and the Amhara region, respectively [16, 24]. In the present study, the cock‐to‐hen ratio was 1:3.97. This was comparable with the findings of other scholars; the overall cock‐to‐hen ratio was 1:3.7 from the Bure district, Northwest Ethiopia, and 1:2.37 from the Benishangul‐Gumuz region [16, 21].

The chicken handling system practiced in this study area was under a traditional production system. They were free‐range scavengers without proper management. It agrees with the report from the southern part of Ethiopia [25]. Chicken management is critical in chicken production [26]. In the current study, chicken management responsibilities within the household were shared by all family members, children, and women. It was comparable to the report of Bogale et al. from Southwestern Ethiopia, where chicken production management activities are mainly shared by all family members (56%) [27]. In contrast to this result, many scholars from different parts of the country reported that the major responsibilities of chicken handling in the household were for women [21, 28]. However, in village‐based chicken production, chicken owners do not examine and manage their chickens properly [11].

More than half of the respondents restock their chickens by hatching at home. This is similar to a study conducted in Chagni town, Awi administrative zone, where farmers depend on broody hens to restock their chickens [24]. Regarding chicken housing, they were confined at night only, and 67.4% of the respondents constructed a separate house for their chickens to rest at night. This is comparable to the report of Mamo et al. from Northern Gondar, where 63% of the chickens rest at night in a separate chicken house [29]. However, Kebede et al. from Northwest Ethiopia reported that only 29.33% of the respondents had constructed a separate traditional chicken house [28]. About 25.6% of the respondents’ chickens share the main house to rest at night (Table 3). This is better than the report of Yitbarek et al. from North Gondar, where 58% of the chickens share the same room with the main house [30]. About 93% of the respondents clean the chickens’ house every day. The result agrees with the report of Leta and Bekana from Oromia, where 81% of the households cleaned chicken houses once per day [31].

In the current study, most of the respondents know about ND, and the high occurrence of ND aligns with previous Ethiopian village poultry studies reporting endemic circulation of ND virus in the country [32–35]. Moreover, Dinka et al. from Oromia reported that ND was identified as a major and economically important chicken health constraint in Ethiopia [36]. However, in other studies from Kisii county in Kenya, the disease has occurred in only 29.3% of the small‐scale poultry farmers [37]. This might be associated with inadequate biosecurity practices in village chicken production. The occurrence of ND was statistically associated with chicken handling practices in the area, such as the disposal practices of dead chickens, contact with neighboring household flocks, and mixing of purchased chickens with their own. This finding is comparable with the report of many scholars. Contact with chickens at the market and neighborhood may serve as a source of infection. In addition, the mixing of chickens of different origins and overcrowding at live chicken markets in the village chicken production systems serve as a focal source for the ND virus maintenance and spread [38–40].

In the current study, about half of the respondents isolate infected chickens and look for veterinary services to treat them. In contrast to this finding, Leta and Bekana reported that only 11.6% of the respondents consult veterinarians when their chickens get sick. All affected chickens had died in about 40% of the households, and in another study conducted in the Oromia region, 67% of sick chickens had died [31]. The high mortality of chickens indicates the presence of virulent field strains in the area. About 41.7% of the households bury dead chickens as a biosecurity measure. However, only 34.5% of poultry farmers from Kisii bury dead birds [37], and most of the respondents (86.8%) from Southern Ethiopia gave dead birds to pet animals [41]. Regarding dead chicken disposal, other than burying, they are thrown out of a compound or into a river or left to be eaten by dogs and cats. This kind of free disposal of dead chickens favors the spread of the NDV, causing an uninterrupted cycle of ND infection [38].

Most of the respondents in this study treat all the chickens available in the household. However, in another study from Southwestern Ethiopia, 35.7% and 55.7% of the respondents treated all their chickens and only sick chickens, respectively [27]. Fourteen percent of the respondents treat their chickens by themselves without consulting a veterinarian. This may play a significant role in antimicrobial resistance development. Nowadays, antimicrobial resistance is increasing alarmingly, and there is a fear of a post‐antibiotic era [42]. In addition, 26.7% of households use medical drugs for the treatment of their chickens. In another study from the southern part of Ethiopia, about 71.7% of smallholder farmers from Sidama have a practice of using conventional medical drugs, such as tetracycline and ampicillin, to treat their chickens [43]. This underscores the importance of awareness creation on proper chicken handling and antimicrobial utilization for improving their chicken production and reducing the development of antimicrobial resistance. In this study, the highest disease occurrence was observed in the summer (42.7%) compared to other seasons. It is in line with the reports of Bogale et al, where most chicken disease outbreaks that cause the loss of their chickens occur in the summer [27]. In addition, another scholar from the Halaba district reported that the severity of the disease was higher in the rainy season (75.4%) than in the dry season [41].

In the current study, the backyard chickens were free scavengers. They were primarily managed under a scavenging feeding system. This finding is comparable to the report of Mohammed, where the village‐based chicken production system in Ethiopia is usually characterized by keeping chickens under a free‐range system [11]. Feed scarcity was aggravated in the wet season (90.5%) compared to the dry season. It is comparable to the report of Yirgu et al. from Southern Ethiopia, where 89.1% of the respondents faced feed scarcity in the wet season [44]. The major grains supplemented to the chickens in the study area were wheat (96.5%) and maize (82.6%). However, only 17.6% of households supplement their chickens with maize in Southern Ethiopia [25]. Most of the respondents feed their chickens in the morning and late afternoon. This result was in line with the report of Kebede et al. from Northwestern Ethiopia, where 69% of the respondents supplement their chickens in the morning and late afternoon [28]. However, only 42.16% of the respondents supplement their chickens in Southwestern Ethiopia [27]. The main reason for supplementation was to improve egg production (65.7%) and both growth and egg production (27.1%). However, another scholar from the Metekel zone, Northwest Ethiopia, reported that only 18.8% of respondents supplement chickens to improve egg production, while 65.4% supplement for egg and meat production [45]. Additionally, Bogale et al. from Southwestern Ethiopia reported that 30.4% and 34.1% of the respondents supplement their chickens to increase egg production and meat and egg yield, respectively [27]. Half of the households (51.8%) use water wells as a source of water for their chickens. However, according to Mamo et al. from North Gondar, the major source of water for chickens was tap water (92%) [29]. Furthermore, Agza from the Metekel zone reported that the major water source for chickens was from rivers (30.5%) [45]. The main predators available in the area were wild cats and eagles. This is comparable to the report of Yirgu et al., where wild cats, eagles, and foxes were the major predators of chickens in Sidama, Ethiopia [43]. Moreover, predation was mainly by wild cats, foxes, and eagles (51.1%) in the Halaba district of Southern Ethiopia [41].

The major constraints identified in the village chicken production in this study were disease and predator attacks. This finding was comparable to the report of Meskerem, where disease and predator attacks were identified as the main causes of chicken loss [46]. Leta and Bekana also reported from the Mid Rift Valley of Oromia that chicken diseases (34%) and predators (34%) were the major threats to village‐based chicken production [31]. In addition, Sambo et al. from Debre Zeit and Abera from the Benishangul‐Gumuz region have reported that a key chicken production constraint identified by chicken farmers was poultry diseases [7, 47]. Moreover, other scholars from North Gondar reported that the main constraints on village chicken production were predation (30%), feed shortage (28%), and flock mortality (28%) [30]. These show the significant impact of diseases and predators on village chicken production in Ethiopia.

## 5. Conclusion

The result of the study indicated that the chickens were managed under a free‐range scavenging system with little grain supplementation. Feed scarcity was aggravated in the wet season compared to the dry season. Poultry disease and predator attacks were the major constraints identified in the area. ND was the major disease in the area, causing high chicken mortality. This might be associated with very poor biosecurity practices, such as dead chicken disposal practices, contact with neighboring household flocks, and the mixing of purchased chickens with their own without quarantine; they did not vaccinate their chicken against common diseases, and they did not practice disease control and prevention biosecurity measures. Therefore, awareness creation for farmers and development agents is required regarding proper chicken handling practices, biosecurity measures, and vaccinating chickens against common diseases, which could reduce mortality, improve the flock size, and improve village chicken production.

## Funding

This study was supported financially by the Ethiopian Institute of Agricultural Research (EIAR), Animal Health Research Program (budget code: 31‐09).

## Conflicts of Interest

The authors declare no conflicts of interest.

## Data Availability

The data that support the findings of this study are available from the corresponding author upon reasonable request.
